# Supplementary material for: Optimal strategies to screen health care workers for COVID-19 in the US: a cost-effectiveness analysis
Source: Res Sq. 2021 Sep 10:rs.3.rs-887590. Preprint. [Version 1] doi: 10.21203/rs.3.rs-887590/v1 (PMC8437316; doi:10.21203/rs.3.rs-887590/v1)
Supplement: Supplement 1 [file 18035649b78a3a6639877743.docx]

**S2 Appendix: RESULTS**

1. **Cost and health outcomes**

Medical costs, screening costs, new infections, and QALYs lost associated with each screening approach are provided in Tables V and VI.

**Table V.** Implementation, medical, and net costs of screening ten HCWs. The lowest cost in each column is in bold. Numbers may not add up due to rounding.

|  |  |  | **Early clinical disease,**  **days 1-7** | |  | **Early clinical disease,**  **days 8-14** | |
| --- | --- | --- | --- | --- | --- | --- | --- |
| **Option** | **Testing costs** |  | **Treatment costs** | **Net costs** |  | **Treatment costs** | **Net costs** |
| **No Test** | **$0** |  | $82,172 | $82,172 |  | $981 | $981 |
| **Only Ag** | $50 |  | $8,243 | $8,293 |  | $303 | **$353** |
| **Only PCR** | $510 |  | $4,123 | **$4,633** |  | $50 | $560 |
| **IgG + PCR** | $930 |  | **$4,107** | $5,037 |  | **$49** | $979 |
| **IgG, if positive PCR** | $460-$648 |  | $63,790 | $64,297 |  | $367 | $836 |
| **Only IgG** | $420 |  | $82,077 | $82,497 |  | $973 | $1,393 |
|  |  |  |  |  |  |  |  |
|  |  |  | **Late clinical disease** | |  | **Asymptomatic** | |
| **Option** | **Testing costs** |  | **Treatment costs** | **Net costs** |  | **Treatment costs** | **Net costs** |
| **No Test** | **$0** |  | $11.4 | **$11.4** |  | $104 | $104 |
| **Only Ag** | $50 |  | **$10.9** | $60.9 |  | $35.9 | **$85.9** |
| **Only PCR** | $510 |  | $11.9 | $52.2 |  | $33.4 | $543 |
| **IgG + PCR** | $930 |  | $10.9 | $941 |  | **$33.1** | $963 |
| **IgG, if positive PCR** | $469-$648 |  | $10.9 | $483 |  | $58.4 | $704 |
| **Only IgG** | $420 |  | $10.9 | $431 |  | $103 | $523 |

**Table VI.** New infections generated and QALYs lost for ten HCWs screened using different screening strategies. The best health outcomes in each column are in bold. Numbers may not add up due to rounding.

|  | **Early clinical disease, days 1-7** | |  | **Early clinical disease, days 8-14** | |
| --- | --- | --- | --- | --- | --- |
| **Option** | **New infections** | **QALYs lost** |  | **New infections** | **QALYs lost** |
| **No Test** | 24.810 | 1.925 |  | 0.296 | 0.02299 |
| **Only Ag** | 2.489 | 0.193 |  | 0.091 | 0.00709 |
| **Only PCR** | 1.245 | 0.097 |  | **0.015** | **0.00117** |
| **IgG + PCR** | **1.241** | **0.096** |  | 0.015 | 0.00116 |
| **IgG, if positive PCR** | 19.260 | 1.495 |  | 0.111 | 0.00860 |
| **Only IgG** | 24.782 | 1.923 |  | 0.294 | 0.02279 |
|  |  |  |  |  |  |
|  | **Late clinical disease** | |  | **Asymptomatic** | |
| **Option** | **New infections** | **QALYs lost** |  | **New infections** | **QALYs lost** |
| **No Test** | 0.00345 | 0.00027 |  | 0.03148 | 0.00244 |
| **Only Ag** | **0.00330** | **0.00026** |  | 0.01093 | 0.00084 |
| **Only PCR** | 0.00362 | 0.00028 |  | **0.01014** | **0.00078** |
| **IgG + PCR** | 0.00661 | 0.00026 |  | 0.01127 | 0.00078 |
| **IgG, if positive PCR** | 0.00330 | 0.00026 |  | 0.01762 | 0.00137 |
| **Only IgG** | 0.00330 | 0.00026 |  | 0.03116 | 0.00242 |
