# Supplementary material for: Optimal strategies to screen health care workers for COVID-19 in the US: a cost-effectiveness analysis
Source: Res Sq. 2021 Sep 10:rs.3.rs-887590. Preprint. [Version 1] doi: 10.21203/rs.3.rs-887590/v1 (PMC8437316; doi:10.21203/rs.3.rs-887590/v1)
Supplement: Supplement 2 [file e0e5656adf382191a6f3ac14.docx]

**S1 Appendix: METHODS**

1. **True disease status**

The true disease status of a HCW depends on the presence or absence of SARS-CoV-2 and antibodies, which is unknown by the individual. The characteristics of the three possible true disease states are shown in Figure 1. *No antibodies* denotes individuals who did not have IgM or IgG antibodies against SARS-CoV-2. These individuals were susceptible to becoming infected, or they had been previously infected and had not developed an immune response, or they were infected and the immune response has waned. *IgM only* pertains to individuals who had produced IgM against SARS-CoV-2 but had not yet developed IgG. Since IgM is the first type of antibody to be produced upon the introduction of a new pathogen, these individuals were likely HCWs who recently became infected and had not yet mounted a full immune response. *IgG only* was used to define HCWs who had produced IgG but did not have IgM. This may occur in individuals who are in the later stages of recovery, since typically IgG is produced after IgM and the titers of the latter wane with time while they remain higher for the former. *Both antibodies* denotes individuals with both IgM and IgG.


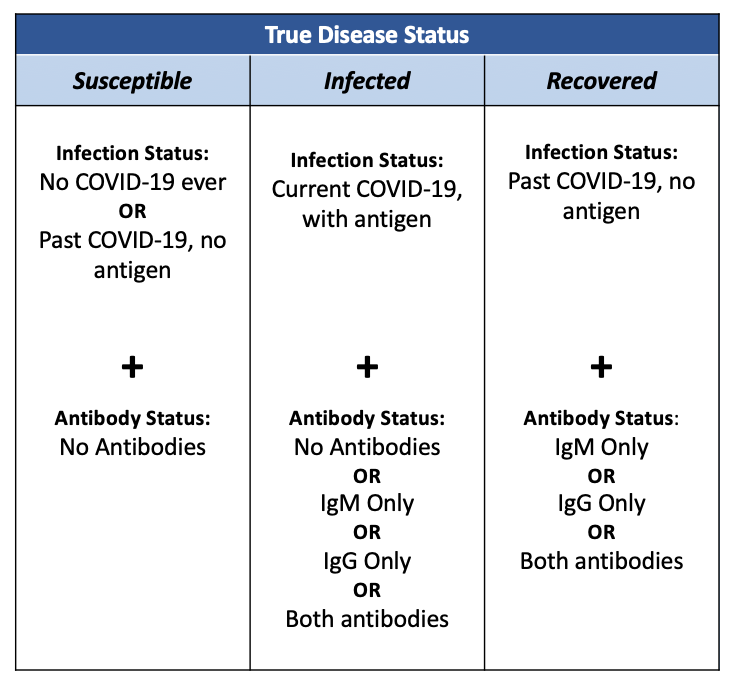


**Figure I.** Infection states and antibody profiles of individuals with different true disease states

1. **Inferred disease status**

Table I outlines the inferences made based on test results. A positive IgG test result indicates recovery in the absence of a positive PCR test. A positive PCR test result generally indicates that the HCW is infected, except the late clinical stage when the likelihood of still having viable virus is so low that these results are interpreted as false-positives and do not induce behavior change. A positive Ag test is always interpreted as transmissible infection.

**Table I.** Inferences of disease status based on test outcomes. Three dots indicate that the test was not conducted.

| **Screening Strategy** | **Ag Test Result** | **PCR Test Result** | **IgG Test Result** | **Inferred Disease Status** |
| --- | --- | --- | --- | --- |
| **No testing** | **…** | **...** | **…** | Unknown |
| **Ag test only** | **+** | **…** | **…** | Infected |
|  | **-** | **…** | **…** | Susceptible |
| **PCR test only** | **…** | **+** | **…** | Infected* |
|  | **…** | **-** | **…** | Susceptible |
| **IgG test only** | **…** | **…** | **+** | Recovered |
|  | **…** | **…** | **-** | Susceptible |
| **IgG test, if positive PCR test** | **…** | **+** | **+** | Infected* |
|  | **…** | **-** | **+** | Recovered |
|  | **…** | **…** | **-** | Susceptible |
| **IgG and PCR tests** | **…** | **+** | **+** | Infected* |
|  | **…** | **-** | **+** | Recovered |
|  | **…** | **+** | **-** | Infected* |
|  | **…** | **-** | **-** | Susceptible |

*Except in the late clinical period where a positive PCR test result is considered a false positive.

1. **Infection control measures**

The guidelines for HCW assignments based on screening results are shown in Table II. The probabilities are not 1 (except for isolation) because of the assumption that it may not be possible to always assign recovered individuals to COVID-19 cohorts and to always assign susceptible individuals to non-COVID-19 units due to scheduling conflicts.

**Table II.** Probabilities of taking the specified infection control measure based on test results.

| **Inferred disease status** | **Isolation*** | **Cold zone^†^** | **Hot zone^¥^** |
| --- | --- | --- | --- |
| *Susceptible* | 0 | 0.8 | 0.2 |
| *Infected* | 1 | 0 | 0 |
| *Recovered* | 0 | 0.2 | 0.8 |
| *Unknown* | 0 | 0.7 | 0.3 |

****Isolation:*** The individual undergoing screening is isolated. ***^†^Cold zone:*** The individual undergoing screening is assigned to a cold zone and does not interact with any COVID-19 patients. They take social distancing and masking precautions in community settings. ***^¥^Hot zone:*** The individual undergoing screening is assigned to a hot zone and only interacts with COVID-19 patients. They are less strict about social distancing and masking precautions in community settings.

1. **Transmissibility**

Table III depicts the transmissibility inputs in base-case. It is assumed that HCWs cannot acquire a second infection of SARS-CoV-2 while infected with active virus and recovered individuals can neither acquire nor transmit infection. When an infected HCW is assigned to a COVID-19 cohort, the likelihood of transmitting the virus to others in the hospital decreases because they would be caring for individuals who are already infected and working alongside other HCWs who have recovered.

**Table III.** Transmissibility characteristics of SARS-CoV-2 given different disease states and infection control measures for the HCW being screened.

The value 1.4 / 100,000 means that each uninfected HCW has a 1.4 in 100,000 risk of acquiring COVID-19 in the community setting over two weeks if isolated (assuming imperfect isolation and 10% the chance of acquisition as being assigned to the cold zone). The value 0.2 means that an infected individual who is isolated will cause 0.2 new infections in the community setting over two weeks (assuming imperfect isolation and 25% the chance of transmission as being assigned to the cold zone).

| **True disease status** | **Probability of acquisition in community setting** | | |  | **# of infections transmitted in community setting** | | |
| --- | --- | --- | --- | --- | --- | --- | --- |
|  | **Isolation*** | **Cold zone^†^** | **Hot zone^¥^** |  | **Isolation*** | **Cold zone^†^** | **Hot zone^¥^** |
| Susceptible | 1.4 / 100,00 | 4.2 / 100,000 | 6.2 / 100,000 |  | 0 | 0 | 0 |
| Infected | 0 | 0 | 0 |  | 0.2 | 0.85 | 1 |
| Recovered | 0 | 0 | 0.6 / 100,000 |  | 0 | 0 | 0 |
|  |  |  |  |  |  |  |  |
| **True disease status** | **Probability of acquisition in health care setting** | | |  | **# of infections transmitted in health care setting** | | |
|  | **Isolation*** | **Cold zone^†^** | **Hot zone^¥^** |  | **Isolation*** | **Cold zone^†^** | **Hot zone^¥^** |
| Susceptible | 0 | 12.4 / 100,000 | 18.7 / 100,000 |  | 0 | 0 | 0 |
| Infected | 0 | 0 | 0 |  | 0 | 1 | 0.8 |
| Recovered | 0 | 1.9 / 100,000 | 2.8 / 100,000 |  | 0 | 0 | 0 |

****Isolation:*** The individual undergoing screening is isolated. ***^†^Cold zone:*** The individual undergoing screening is assigned to a cold zone and does not interact with any COVID-19 patients. They take social distancing and masking precautions in community settings. ***^¥^Hot zone:*** The individual undergoing screening is assigned to a hot zone and only interacts with COVID-19 patients. They are less strict about social distancing and masking precautions in community settings.

1. **PCR test performance**

PCR tests detect a specified portion of RNA but cannot distinguish between a live viral pathogen and fragments of RNA left behind. In this study, we defined the term “infected” based on being infectious. Per this definition, a true positive PCR test result would entail the presence of viable virus only. Even if the test accurately detects viral fragments, this would be considered a false positive since the HCW tested would no longer be infectious. To reflect this, we adjusted PCR performance for time since symptom onset.

In days 1-7 post-symptom onset, being infected and being infectious are closely aligned; it is very likely that an infected individual still has viable virus and can infect others. At this time period, PCR test performance for infectivity should reflect the marketed test performance and have high sensitivity and specificity.

Research suggests that viral cultures could not be obtained approximately 8 to 11 days after symptom onset, indicating that the alignment of infection versus infectivity is lost sometime in the second week post-symptom onset.(6,11,20) While a false negative is still unlikely (and sensitivity remains high), this suggests that a positive PCR result obtained in days 8-14 (or thereafter) is likely due to viral fragments and should be considered a false positive in the context of our analysis. Xiao et al reports an 89.3% PCR-positivity rate in a sample of 56 patients known to be infected with SARS-CoV-2 in days 8-14 post-symptom onset.^40^ Within this timeframe, 7.9% of infected individuals are assumed to remain infectious.^17^ Using these values and 95% sensitivity:

$$Specificity (days 8-14) = \frac{\% not infectious - (\% test positive - \% true positive)}{\% not infectious} = \frac{92.1 - (89.3 - 0.95x7.9)}{92.1} = 0.11$$

The upper and lower bounds of specificity were calculated using the upper and lower bounds for PCR sensitivity and applying the same formula.

15-39 days after symptom onset, virtually no viable SARS-CoV-2 remains in infected individuals.(21) Consequently, the sensitivity becomes irrelevant. The body also continues to eliminate the remaining viral fragments, so specificity once again increases. Using 34.5% PCR positivity rate and applying the formula above, we estimated the PCR specificity to be 0.66 at this point in the course of disease.(48)

The specificity value used for the strata of asymptomatic HCWs is an arithmetic average of the specificities estimated for the three other strata.

1. **Cost of COVID-19 treatment**

The cost of treating COVID-19 depends on severity. While asymptomatic individuals will not require any treatment and generate no costs, severely ill individuals require critical care and incur very high costs. We used the distribution of disease severity among those infected with COVID-19 and the respective average costs incurred for each level of severity to calculate a weighted average cost of treatment (Table IV).(31)

**Table IV.** Breakdown of treatment costs by type of care sought.(31)

ED: emergency department; ICU: intensive care unit.

| **Type of care needed** | **% of infected** | **Cost** |
| --- | --- | --- |
| Does not seek care | 79% | $0 |
| Seeks outpatient care | 2% | $513 |
| Seeks care at ED | 4% | $815 |
| Requires hospitalization | 12% | $12,000 |
| Requires ICU admission | 3% | $61,000 |
| **Weighted total** | **100%** | **$3,313** |

1. **Decision tree**

A simplified version of the decision tree is presented in Figure II.


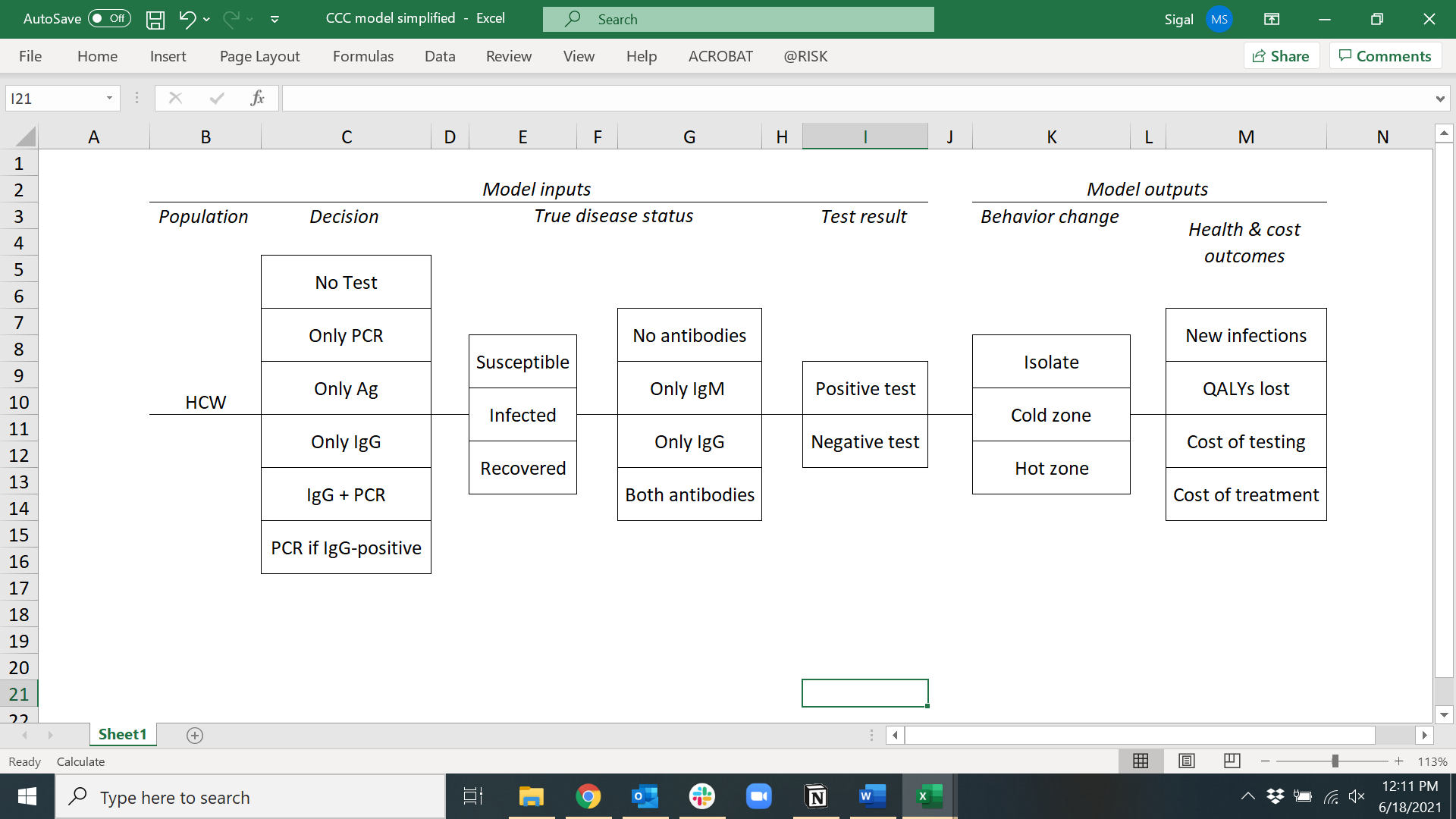


**Figure II.** Simplified decision tree.
